# Recombinase polymerase amplification assay combined with a lateral flow dipstick for rapid detection of *Tetracapsuloides bryosalmonae*, the causative agent of proliferative kidney disease in salmonids

**DOI:** 10.1186/s13071-018-2825-5

**Published:** 2018-04-11

**Authors:** Hatem Soliman, Gokhlesh Kumar, Mansour El-Matbouli

**Affiliations:** Clinical Division of Fish Medicine, University of Veterinary Medicine, Veterinärplatz 1, 1210 Vienna, Austria

**Keywords:** Diagnosis, PKD, Isothermal amplification, RPA, Trout

## Abstract

**Background:**

The myxozoan *Tetracapsuloides bryosalmonae*, the causative agent of proliferative kidney disease (PKD), is responsible for considerable losses in farmed and wild fish populations in Europe and North America. Recently, *T. bryosalmonae* was detected in many European countries, and strategy to control the disease in the wild and farmed fish population is yet to be developed. Recombinase polymerase amplification (RPA) is a novel isothermal nucleic acid amplification technology that does not require any thermal cycling, and lateral flow dipstick (LFD) is a rapid, cost-effective, and easy-to-handle assay that enables stable detection.

**Results:**

In this study, we developed and optimized a rapid and sensitive RPA assay combined with an LFD for the detection of *T. bryosalmonae*. The PKD-RPA assay was specific to *T. bryosalmonae*, as no cross-reaction or false positive signals were observed with any of the other tested DNAs. The developed PKD-RPA assay was ten times more sensitive than an existing diagnostic polymerase chain reaction (PCR) assay for this parasite. The estimated time to perform PKD-RPA assay is 25 min compared to 4 h for PKD-PCR assay.

**Conclusions:**

A novel PKD-RPA assay for the detection of *T. bryosalmonae* was developed. The assay offers considerable advantages including speed, sensitivity, specificity and visual detection. Applying the PKD-RPA assay combined with an LFD enhances the surveillance and early detection of *T. bryosalmonae* in salmonids.

## Background

Proliferative kidney disease (PKD), an emerging parasitic disease, is threatening both farmed and wild salmonid populations in North America and Europe [1, 2]. The malacosporean parasite *Tetracapsuloides bryosalmonae* is the causative agent of PKD in salmonids [3]. *Tetracapsuloides bryosalmonae* uses bryozoan *Fredericella sultana* as invertebrate hosts and salmonids as vertebrate hosts to complete its life-cycle [4–6]. The water-borne *T. bryosalmonae* spores, which are released from infected bryozoan colonies, enter through the gill epithelium [7, 8] and reach the kidney through the vascular system [9], where further propagation and differentiation occurs. Mature spores are excreted in urine to infect the bryozoan host [4, 5]. Water temperature plays a crucial role in the development and pathology of PKD in salmonid fish, as a direct positive relationship exists between the severity of clinical signs, as well as associated mortality, and increasing water temperature [1, 10–12]. The clinical symptoms of PKD in fish are exophthalmia, enlarged kidney, splenomegaly, darkened body, pale gills and abdominal distension [1]. The mortality rates vary and can reach 100% when secondary infections are involved [13–15]. In some European rivers, PKD is widely distributed and assumed to be responsible for mortalities in wild salmonid populations, particularly brown trout *Salmo trutta* and Atlantic salmon *Salmo salar* [16–21]. Multiple dispersal routes likely contribute to distribution of PKD parasite such as vertical transmission of *T. bryosalmonae* in statoblasts [22, 23] and escaping of migrating zooids from deteriorating bryozoan colonies [24]. The molecular analysis of *T. bryosalmonae* ITS1 strains has revealed two main lineages, originating from Europe and North America [25]. Traditionally, a presumptive diagnosis of PKD was made by the detection of the *T. bryosalmonae* spores in the kidney using wet mounts, Giemsa-stained or lectin-stained kidney imprints [26–28]. The confirmatory diagnosis was based on the histological examination of tissue sections stained with hematoxylin and eosin [29–31]. In addition, specific monoclonal antibodies were used for the detection of *T. bryosalmonae* in the infected fish kidney [32–34]. Subsequently, DNA-based detection assays, such as *in situ* hybridization, polymerase chain reaction (PCR), real-time PCR and loop-mediated isothermal amplification assays, were developed for specific and sensitive detection of *T. bryosalmonae* [35–43]. Despite its advantages, PCR has intrinsic disadvantages, such as time-consuming, expensive instruments, and complicated procedures for the detection of amplified products [44, 45].

Recombinase polymerase amplification (RPA) is a novel isothermal nucleic acid amplification technology that does not require any thermal cycling, as the amplification reaction is performed at a constant low temperature [46]. Notably, RPA utilizes an enzymatic mixture of polymerase and DNA recombination proteins and can be performed using a simple shaking incubator [46, 47]. Recombinase enzyme binds to the primers to form a complex that promotes primers to anneal to its homologous sequence in a double-stranded DNA (dsDNA) template; the DNA polymerase then displaces the dsDNA strands and elongates the primer, resulting in an exponential amplification of the target [46, 48–50]. By including a specific probe into the RPA reaction solution, the amplification products can be visualized by a lateral flow dipstick (LFD), which is more advantageous than the other detection methods because of its rapidity, low-cost, ease of use and long-term stability [51, 52].

This study aimed to use RPA assay for isothermally amplifying *T. bryosalmonae* DNA from *T. bryosalmonae*-infected fish, and for detecting amplified products using LFD. The sensitivity and reliability of this detection method were assessed and compared with those of conventional diagnostic PCR.

## Methods

### Sample collection and DNA extraction

Specific pathogen-free (SPF) and *T. bryosalmonae*-infected *F. sultana* colonies were maintained in a laboratory culture system following methods of Kumar et al. [53]. Approximately 35 zooids were collected from SPF *F. sultana* colonies and subjected to DNA extraction to be used as a negative control. *T. bryosalmonae* mature spore sacs (*n* = 40) were collected from infected *F. sultana* colonies and subjected to DNA extraction to be used as a positive control. Kidney tissues from both SPF and *T. bryosalmonae*-infected brown trout that were sampled in our previous study [54] with ethical permission (GZ 68.205/0247-II/3b/2011) were subjected to DNA extraction. All DNA extractions were performed using DNeasy blood and tissue kit (Qiagen, Hilden, Germany) according to the manufacturer’s instructions. DNA concentrations were measured using NanoDrop 2000 spectrophotometer (Thermo Fisher Scientific, Vienna, Austria) and then stored at -20 °C until use.

### PKD-RPA primers and probe design

The primers and probe for PKD-RPA assay (Table 1) were designed to amplify a 141 bp segment of internal transcribed spacer 1 (ITS1) sequence of the rRNA gene of *T. bryosalmonae* (GenBank accession number JQ424926). All the available *T. bryosalmonae* ITS1 sequences from the National Center for Biotechnology Information (NCBI) database were aligned, and a highly conserved region was selected and used for designing the PKD-RPA primers and probe according to the TwistDX (TwistDx Ltd., Cambridge, UK) guidelines. The forward primer (PKD-RPA_F) was a normal primer (unlabeled), and the reverse primer (PKD-RPA_R) was labeled with digoxigenin at the 5' end. The probe (PKD-RPA_P) was labeled with 6-carboxyfluorescein (FAM) and a polymerase extension blocking group (C3-spacer) at its both 5' and 3' ends, respectively. In addition, the probe was modified internally by replacing one nucleotide with tetrahydrofuran (THF; also known as dSpacer). The specificity of the primers and probe was assessed *in silico* using the basic local alignment search tool (BLAST) against the NCBI nucleotide database to ensure that no homology exists with other organism sequences. The primers and probe were synthesized by Eurofins Genomics (Ebersberg, Germany).

**Table 1:** The oligonucleotides used in this study

Primer name	Primer sequence (5'–3')	Genome position	Primer length (bp)	GenBank ID	Method	Reference
PKD-RPA_F	TAATACGGATGTGGGTTAGTGGAAACTGGG	48–77	30	JQ424926	RPA	This study
PKD-RPA_R	Digoxigenin-CGTGAATCAGACCAAATATCTTCAAGATGACAAG	155–188	34			
PKD-RPA_P	FAM-GGAGTGGGAAGATAATACGTAAACGTGCATTZGCCAAGTATTTAATAG-(C3spacer)	106–153	48			
PKX 5 F	CCTATTCAATTGAGTAGGAGA	463–483	21	U70623	PCR	[36]
PKX 6 R	GGACCTTACTCGTTTCCGACC	877–897	21			

### PKD-RPA assay

The PKD-RPA reaction was performed using a TwistAmp nfo kit (TwistDx Ltd.). Various reaction temperatures, incubation times, and primer concentrations were tested to determine the optimal reaction conditions. The PKD-RPA reaction was carried out in a total volume of 50 μl containing 3 μl PKD-RPA_F primer (10 μM), 3 μl PKD-RPA_R primer (10 μM), 1 μl PKD-RPA_P probe (10 μM), 29.5 μl rehydration buffer, 11 μl of both DNA template and nuclease-free water, and finally, one enzyme pellet from the kit was added to each reaction tube. To start the reaction, 2.5 μl of 280 mM magnesium acetate was added and then the tubes were incubated at 38 °C for 4 min. The reaction mixtures were then mixed briefly and re-incubated at the same temperature for another 16 min. PKD-RPA amplification products were detected by the visual inspection of a colorimetric signal on a nucleic acid LFD (HybriDetect 2T; Milenia Biotec, Giessen, Germany). PKD-RPA amplification products (2 μl) were mixed with 98 μl of assay buffer [1× phosphate buffered saline (PBS), 0.1% Tween 20; PBS-T]. Subsequently, 10 μl of diluted RPA product was pipette directly into the sample application area of the LFD, which was then placed in 200 μl PBS-T buffer for 5 min at room temperature. When the test and control lines were simultaneously visible, it was considered positive. In addition, if only the control line was visible, it was considered negative. The control line confirmed that the LFD was working correctly. All tests were performed under the same conditions in triplicate.

### Specificity of the PKD-RPA assay

The PKD-RPA assay was evaluated by cross-reaction tests using a range of DNA templates, including those from *Buddenbrockia plumatellae* (our own sample that was confirmed with PCR and sequencing), *Myxobolus cerebralis*, *T. bryosalmonae*, SPF *F. sultana*, *T. bryosalmonae-*infected *F. sultana*, and kidney tissues from SPF and *T. bryosalmonae*-infected brown trout. All these templates were tested in triplicate under the optimal conditions determined for PKD-RPA assay.

### Sensitivity of the PKD-RPA assay

The lower detection limit of the PKD-RPA assay was determined using 10-fold serial dilutions of DNA (10 ng) from pure *T. bryosalmonae* sacs with and without prior mixing with DNA (100 ng) from SPF brown trout kidney. The detection limit of PKD-RPA assay was compared with that of commonly used PCR assay for the detection of *T. bryosalmonae* DNA developed by Kent et al. [36]. All the reactions were performed in triplicate according to the determined optimal conditions for PKD-RPA and PCR assays. The detection limit was defined as the lowest concentration to test positive in the triplicate assays.

### Applicability of the PKD-RPA assay

After optimization of the reaction conditions for the PKD-RPA assay, the feasibility of using this assay for the detection of *T. bryosalmonae* DNA in clinical samples was evaluated by testing retrospective DNA samples that were extracted from 86 clinical fish samples (brown trout and rainbow trout), that had been submitted to our clinic for PKD diagnosis. The results of the PKD-RPA assay were compared with those of the commonly used diagnostic PCR assay [36].

## Results

### Optimization of the PKD-RPA assay

**Fig. 1:**
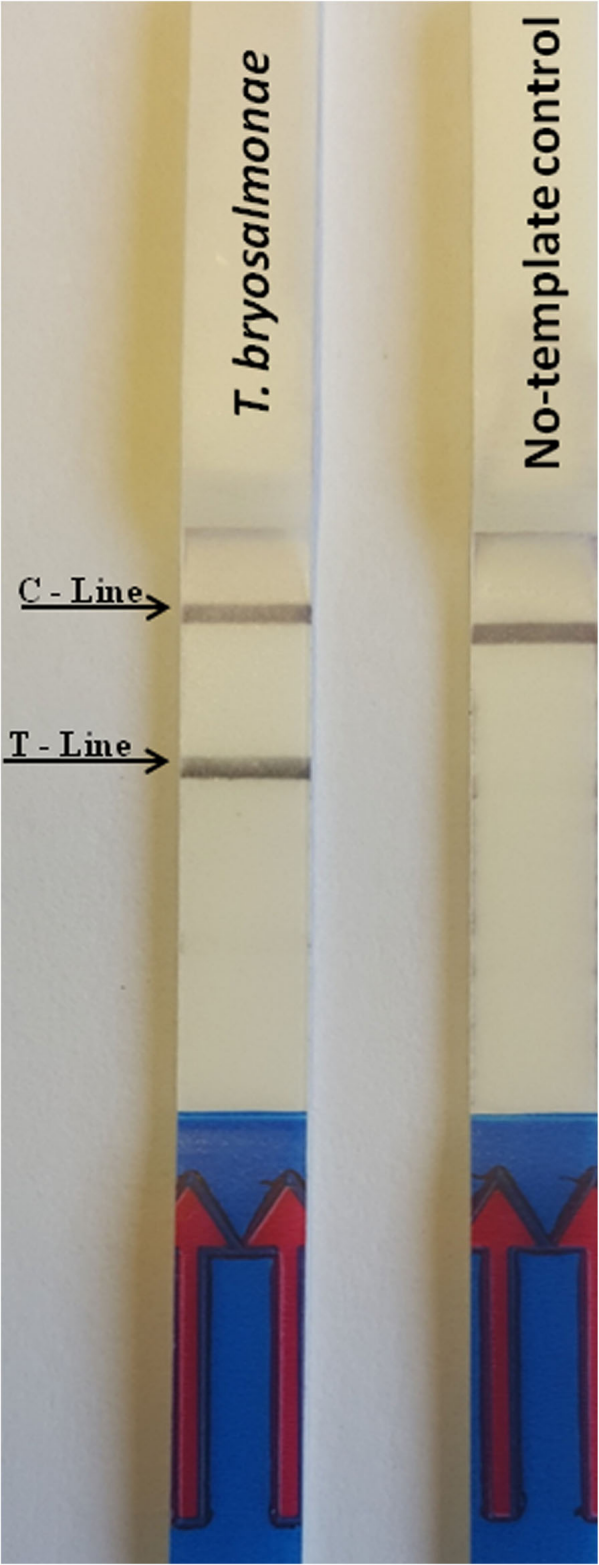
PKD-RPA assay. The color signals on the lateral flow dipstick (LFD), indicating the amplification of *Tetracapsuloides bryosalmonae* DNA using specific recombinase polymerase amplification (RPA) primers and probes. C-line: control signal, which indicates that the LFD functioned correctly; T-line: location of the signal of amplified *T. bryosalmonae* DNA

The PKD-RPA assay functioned at a wide range of temperatures. No differences in amplification were observed when the PKD-RPA assay was performed between 36 °C and 46 °C. However, no amplification signals were detected when the reaction was performed at room temperature or 50 °C. The evaluation of the PKD-RPA amplification time revealed that obvious test bands could be observed on the LFD when the reaction mixture was incubated for 10, 15, 20, 25 and 30 min; however, no signal was detected at 5 min (data not shown). Considering the detection efficiency, sensitivity and rapidity, the reaction temperature and amplification time were selected as 38 °C and 20 min, respectively, for all subsequent experiments. Under the optimized conditions, the PKD-RPA assay results demonstrated that the designed primers (30 pmol/reaction each) and probe (10 pmol/reaction) can amplify the target *T. bryosalmonae* DNA, while, no test bands were observed on the LFD when no-template control was used (Fig. 1).

### Specificity and sensitivity of the PKD-RPA assay

**Fig. 2:**
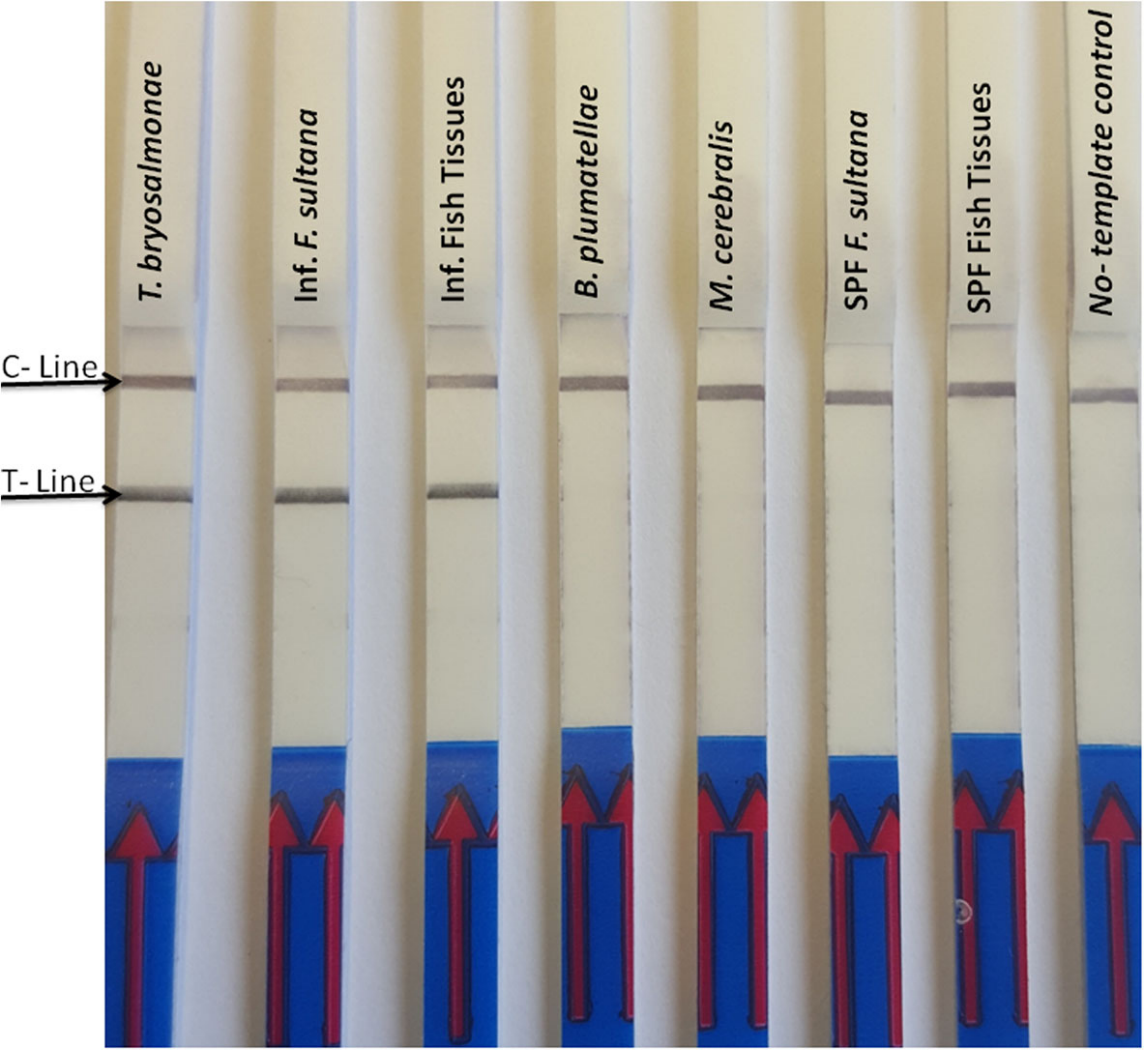
Specificity of the PKD-RPA assay. Specific amplification of *T. bryosalmonae* DNA only is indicated by a color signal on both control and test lines for *T. bryosalmonae* positive samples only, and not with other tested DNA (which show only one color signal, on the control line). C-line: control signal; T-line: location of *T. bryosalmonae* amplicon; Lane *T. bryosalmonae*: DNA from pure *Tetracapsuloides bryosalmonae* mature spore sacs; Lane Inf. *F. sultana*, DNA from *T. bryosalmonae* infected *Fredericella sultana* colonies; Lane Inf. fish tissues: DNA from *T. bryosalmonae* infected fish kidney; Lane *B. plumatellae*: DNA from confirmed *Buddenbrockia plumatellae* sample; Lane *M. cerebralis*: DNA from *Myxobolus cerebralis* spores. Lane SPF *F. sultana*: DNA from specific pathogen-free *Fredericella sultana colonies*; Lane SPF fish tissues: DNA from specific pathogen-free fish kidney; Lane No-template control: DNA was omitted from the reaction

The evaluation of the specificity of the PKD-RPA assay revealed that no cross-reaction or false positive signals were observed with any of the other tested DNA samples (Fig. 2). Furthermore, *in silico* analysis of the primer and probe set designed for the PKD-RPA assay indicated 100% identity to the ITS1 sequences of *T. bryosalmonae* with 100% query coverage.

**Fig. 3:**
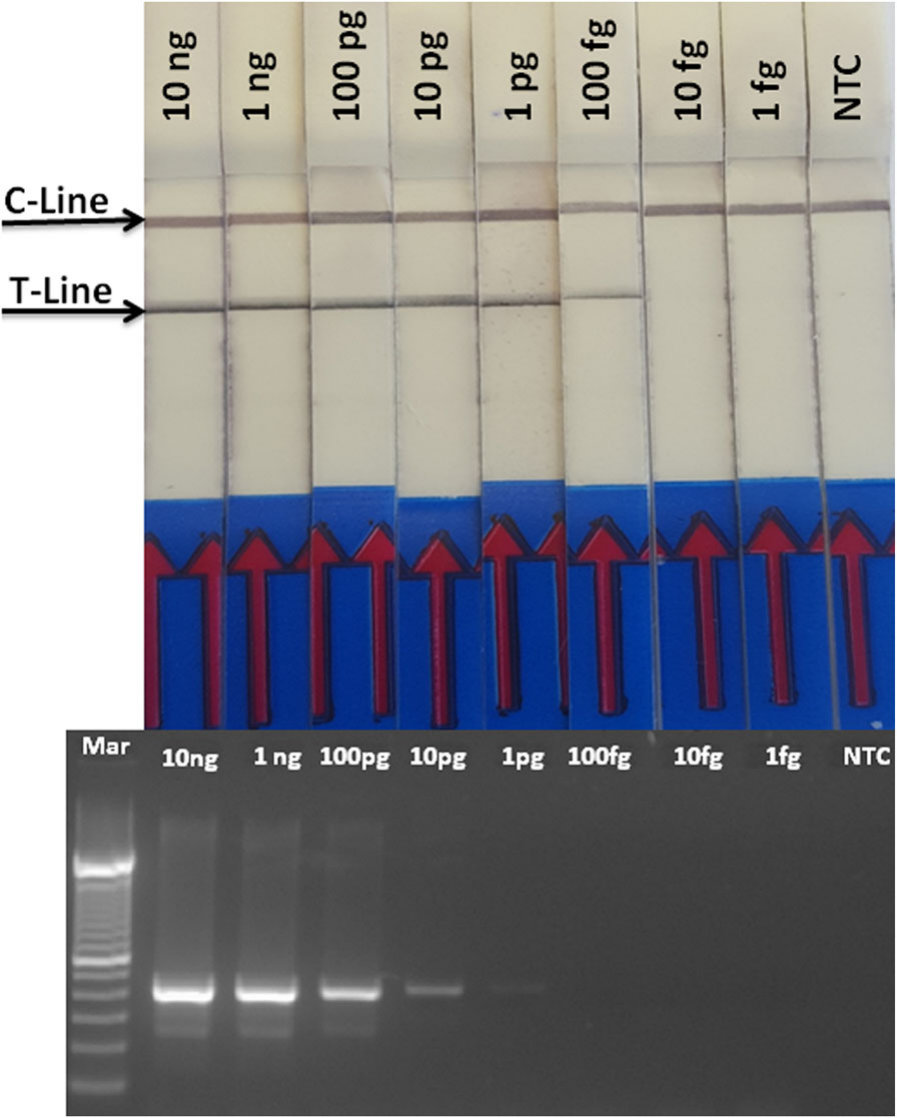
Sensitivity of the PKD-RPA assay compared with the PCR assay. The figure shows the lower detection limit of the developed PKD-RPA assay compared with the diagnostic PCR assay. Ten-fold serial dilutions of pure DNA from *T. bryosalmonae* spore sac (10 ng) was used for this purpose. The PKD-PCR and PKD-RPA assays could detect down to 1 pg and 100 fg of *T. bryosalmonae* DNA, respectively, which is indicated by the presence of a PCR band at 435 bp and the presence of two color signals on the LFD, respectively. C-line: control signal; T-line: location of PK -amplicon; Lane Mar: 100 bp DNA ladder (Invitrogen); Lane NTC: No-template control

The PKD-RPA assay had a lower detection limit of 100 fg for *T. bryosalmonae* DNA in the presence or absence of genomic DNA of the host, which is 10-times more sensitive than that of the conventional PCR assay (Fig. 3).

### Applicability of the PKD-RPA assay

The applicability of the PKD-RPA assay in detection of *T. bryosalmonae* DNA was evaluated using DNA from 86 clinical fish samples against the conventional diagnostic assay as a reference. The PKD-RPA assay accurately detected *T. bryosalmonae* DNA in the samples that had previously tested positive by PCR assay (38 samples); however, no obvious test bands could be observed on the LFD for the remaining samples (48 samples) that had previously tested negative by PCR assay, with no false positive or negative.

## Discussion

*Tetracapsuloides bryosalmonae* was detected in wild fish species throughout Europe [16, 18–21, 43, 55]. Generally, most of the microbial detection methods are based on PCR and real-time PCRs. Despite their several advantages, these techniques (PCR and real-time PCR) have drawbacks that severely limit their suitability for point-of-use detection and have led to the development of alternative amplification methods [44, 45]. Currently, isothermal amplification methods have been developed to overcome these drawbacks and their features greatly simplify the implementation of these methods in point-of-use diagnosis [45]. Hence, we developed the current assay on the basis of the RPA technique for the rapid, specific, isothermal detection of *T. bryosalmonae* DNA in fish tissues. The first step in the development of a nucleic acid-based detection assay is to identify and obtain the target sequence [56]. The ITSs are associated with the rRNA gene, which lie between the small subunit ribosomal RNA and the large subunit ribosomal RNA coding regions [57]. ITSs are highly variable and, therefore, may be used for species differentiation [58] or even for isolate differentiation among some parasites [59]. This is the same gene (rRNA) target that is used in the PKD-PCR assay and thus provided a measure of direct comparison between PCR and PKD-RPA assay sensitivity. The PKD-RPA primers were 30 and 34 bp, which is approximately the minimum length required by the recombinase protein for the incorporation of oligonucleotides into duplex DNA [46]. We designed the PKD-RPA primers to generate only a short amplicon (141 bp) that would be produced in a relatively shorter time, thus improving the speed of the assay [60]. The use of an LFD for the detection of amplification products increases the specificity of test results and further decreases the total time for the RPA assay [61]. The high degree of LFD specificity was attributed to the use of gold-labeled anti-FAM antibodies to specifically capture RPA products, which were detected by a colorimetric signal [62]. The colorimetric signal can be detected by the naked eye, which not only saves time but also eliminates the need for any other post-amplification detection protocol and trained personnel to interpret the results [63].

We selected 38 °C for 20 min as the optimum amplification conditions for enzyme performance and speed, without reducing the sensitivity of the assay. Knowing that the assay can perform well outside these values makes it robust and flexible and promotes the more successful application of the RPA diagnostic test [64].

The results have demonstrated that the analytical sensitivity of the PKD-RPA assay was 10-times more sensitive than that of a diagnostic PCR developed by Kent et al. [36] and 10-times less sensitive than the real-time PCR assay developed by Fontes et al. [43] (data not shown). Furthermore, compared with PCR and real-time PCR, RPA has some fundamental advantages, such as the ability to function in the presence of several known PCR inhibitors [65, 66]. In addition, the crowding agent in the RPA formulation, high molecular weight polyethylene glycol, enhances the enzyme catalytic activity and improves sensitivity, efficiency, and specificity of the reaction [67–70]. Moreover, the PKD-RPA assay can be performed much faster (25 min) than the PKD-PCR assay and post-amplification analysis (4 h). The current cost of the RPA (reaction and detection) is approximately €5 per test, which is quite high compared with other molecular detection assays. However, prices are likely to decrease in the future while availability and throughput will increase.

RPA can specifically detect DNA in a shorter time than any other nucleic acid detection methods and is tolerant to impure samples [63]. The diagnostic validation of the PKD-RPA assay with the 86 clinical samples revealed 100% specificity and sensitivity. Accordingly, PKD-RPA is considered an accurate and rapid assay for field screening and mass monitoring of PKD in fish.

## Conclusions

We developed a novel PKD-RPA assay for the detection of *T. bryosalmonae* that offers considerable advantages including rapidity, sensitivity, specificity and visual detection. These advantages make PKD-RPA a promising assay for the field monitoring of PKD in salmonids.

## Abbreviations

BLAST: Basic Local Alignment Search Tool
dsDNA: double-stranded DNA
FAM: 6-carboxyfluorescein
ITS1: Internal transcribed spacer 1
LFD: Lateral flow dipstick
NCBI: National Center for Biotechnology Information
PBS: Phosphate-buffered saline
PCR: Polymerase chain reaction
PKD: Proliferative kidney disease
RPA: Recombinase polymerase amplification
rRNA: ribosomal RNA
SPF: Specific pathogen-free
THF: Tetrahydrofuran
